# Computational drug–drug interaction prediction mediated by CYP450 isoforms of Ilaprazole coadministered with clopidogrel

**DOI:** 10.2144/fsoa-2023-0277

**Published:** 2024-05-20

**Authors:** Priyadharshini Ananthathandavan, Damodharan Narayanasamy

**Affiliations:** 1 Department of Pharmacy Practice, SRM College of Pharmacy, SRM Institute of Science & Technology, Kattankulathur-603203, Chengalpattu, Tamilnadu, India; 2 Department of Pharmaceutics, SRM college of Pharmacy, SRM Institute of Science and Technology, Kattankulathur-603203, Chengalpattu, Tamilnadu, India

**Keywords:** clopidogrel, CYP2C19 inhibition, drug–drug interaction, Ilaprazole, *in silico* study

## Abstract

Ilaprazole due to its pharmacokinetic variability does not affect the clopidogrel efficacy during concomitant use. **Methodology:** Prediction of DDI for Clopidogrel and PPIs performed using (DDI-Pred) Way2Drug software. The probabilities ΔP, which estimate the potential DDIs resulting from interaction with CYP450 isoenzymes. **Results:** Positive ΔP-values for CYP2C19 (0.955) indicate that it is involved in the drug interaction of Ilaprazole and Clopidogrel. **Discussion:** Pantoprazole and Ilaprazole were found to have a low probability of CYP2C19 inhibition **Conclusion:** Compared with other PPIs, Pantoprazole and Ilaprazole were found to have a low probability of CYP2C19 inhibition; Since Ilaprazole has pharmacokinetic variability, further *in vivo* and *in vitro* studies are required on the ilaprazole and clopidogrel combination to assess the effect of drug–drug interaction.

## Abstract (plain language summary)

Proton pump inhibitors (PPIs) are generally metabolized by CYP2C19 and CYP3A4 isoenzymes, thereby affecting the efficacy of clopidogrel when administered concomitantly.

The pharmacokinetic variability of ilaprazole seems less than that of other PPIs. CYP2C19 is not a significant factor contributing to the metabolism of ilaprazole as it is metabolized mainly by CYP3A4/3A5 isoforms.

Ilaprazole might be the new substitute as other PPIs are found to be potent CYP2C19 inhibitors.

PASS software predicts the drug–drug interactions (DDI) related to the induction or inhibition of drug-metabolizing enzyme (DME) mediated by cytochrome P450 isoenzymes.

This study aimed to predict metabolic-related DDI mediated by Cytochrome P450 isoforms in ilaprazole coadministered with clopidogrel compared with other proton pump inhibitors.

The drug interactions on the level CYP450 have been predicted for ilaprazole and clopidogrel, and found that it has also been carried out by CYP2C19 metabolic enzyme.

Compared with other PPIs, Pantoprazole and Ilaprazole were found to have a low probability of CYP2C19 inhibition as per *in silico* results. There is insufficient evidence regarding which PPI among pantoprazole and ilaprazole is least likely to interact when coadministered with clopidogrel.

Since Ilaprazole has pharmacokinetic variability, further *in vivo* and *in vitro* studies are required on the ilaprazole and clopidogrel combination to assess the effect of drug–drug interaction.

Clopidogrel is an antiplatelet drug indicated for preventing and treating ischemic vascular disease. It is a prodrug converted to its active metabolite via two oxidative steps mediated by liver cytochrome P450 (CYP450) isoenzymes [1]. CYP2C19 contributed in both oxidative steps in converting clopidogrel to its active metabolite, and CYP3A4 contributed majorly in the second oxidative step [2].

Patients on clopidogrel therapy frequently need to take several medications concomitantly. Thus, variations in clopidogrel responsiveness and clinical result may be caused by pharmacokinetics (PK) and pharmacodynamics (PD) level drug–drug interactions (DDI) that impact platelet activation and aggregation and plasma levels of clopidogrel active metabolite [3]. Proton pump inhibitors (PPIs) and clopidogrel were given concomitantly in clinical practice to lower the risk of gastrointestinal (GI) bleeding related to antiplatelet medication [4]. PPIs are also metabolized, particularly by CYP2C19 and CYP3A4 isoenzymes, thereby affecting the efficacy of clopidogrel when administered concomitantly [5,6]. Research studies suggest to avoid prescribing Clopidogrel with CYP2C19 inhibitors. This drug–drug interaction (DDI) does not seem to be class effect and few studies revealed that it might not be enough to take clopidogrel and PPI separately to avoid this interaction [7,8].

Ilaprazole, third-generation proton pump inhibitor, found to have several advantages like prolonged duration of action with lower interindividual variability even with lower dose and fewer times of administration. The pharmacokinetic variability of ilaprazole seems less than that of other PPIs. CYP2C19 is not a significant factor contributing to the metabolism of ilaprazole as it is metabolized mainly by CYP3A4/3A5 isoforms [9]. As compared with other PPIs, ilaprazole has better drug metabolism and pharmacokinetic properties, which are linked to its pharmacodynamics and clinical results [10–12].

Ilaprazole circumvents the drawbacks of other PPIs caused by CYP2C19. It is well acknowledged that a key factor in the clearance of ilaprazole is CYP3A4-mediated metabolism. Ilaprazole might be the new substitute as other PPIs are found to be potent CYP2C19 inhibitors [13].

The computational or *in silico* methods predict drug–drug interactions (DDIs) indirectly. They will be intensively used to reduce investigation costs before initiating *in vivo* and *in vitro* studies, but not replacing them [14]. The way2drug web tool used for prediction of the DDIs mediated by the seven most important P450 cytochromes: CYP1A2, CYP2B6, CYP2C19, CYP2C8, CYP2C9, CYP2D6 and CYP3A4. PASS software predicts the DDI related to the induction or inhibition of drug-metabolizing enzyme (DME) mediated by cytochrome P450 isoenzymes [15,16]. The current study aimed to predict metabolic-related DDI mediated by Cytochrome P450 isoforms in ilaprazole coadministered with clopidogrel compared with other proton pump inhibitors.

## Methodology

Prediction of DDI for clopidogrel and PPIs performed using (DDI-Pred) Way2Drug software (http://www.way2drug.com/PASSonline/). The models for DDIs prediction in this software are based on the Prediction of Activity Spectra for Substances (PASS) technology, general unrestricted structure–activity relationship (GUSAR) program, and Pairs of Substances Multilevel Neighborhoods of Atoms (PoSMNA) sub-structural descriptors.

This assesses the probability of possible DDIs and predicts the metabolic DDI mediated by the isoenzymes of the CYP450 subfamily according to Operational Classification (ORCA). The PASS software can also predict biological activity, comparable to the prediction of drug–drug interactions mediated by cytochrome P450 isoforms. The input data for DDI prediction are pairs of structural formulas or generic names of the drugs. The probabilities ΔP, which estimate the potential DDIs resulting from interaction with CYP1A2, CYP2B6, CYP2D6, CYP2C8, CYP2C9, CYP2C19 and CYP3A4, give the prediction findings for each pair of substances.

## Results

**Figure 1.**
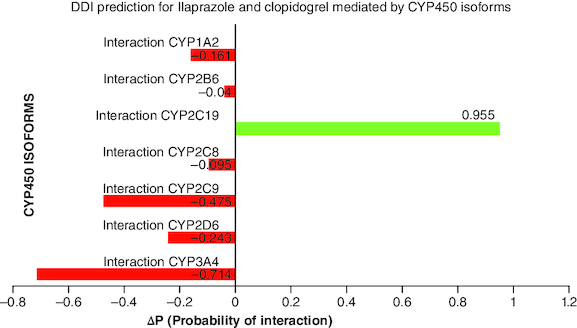
Drug–drug interaction prediction for Ilaprazole and clopidogrel mediated by CYP450 isoforms.

The results of the drug–drug interaction prediction for ilaprazole and clopidogrel mediated by ‘CYP450 Isoforms (PASS double mol) (7 CYP)’ show that the maximum Δp-value (0.955) was calculated for cytochrome P450 CYP2C19 (Figure 1). Therefore, the DDI for clopidogrel and ilaprazole most likely to occur at the level of biotransformation carried out by cytochromeP450 CYP2C19.

Negative ΔP (probability of interaction) values for cytochrome P450 isoforms show that those enzymes are not facilitated in the drug interaction.

Positive ΔP (probability of interaction) values for CYP2C19 indicate that it is involved in the drug interaction.

**Table 1.** Probability of overlapping CYP450 activities of Ilaprazole and Clopidogrel.

CYP450 isoforms	Inhibitor (ΔP)		Substrate (ΔP)		Inducer (ΔP)	
Drug	Clopidogrel	Ilaprazole	Clopidogrel	Ilaprazole	Clopidogrel	Ilaprazole
CYP2C19	0.109	0.72	0.538	0.709	-	-
CYP3A4	-	0.393	0.242	0.593	-	0.722
CYP2C9	0.019	0.561	0.822	0.682	-	-
CYP2D6	-	0.153	-	0.2	-	-
CYP1A2	-	-	-	0.761	-	0.905

**Table 2.** Drug–drug interaction prediction for clopidogrel and various proton pump inhibitors mediated by CYP450 isoforms.

Drug combination	Interaction on isoforms of Cytochrome P450 (ΔP)						
	CYP1A2	CYP2B6	CYP2C19	CYP2C8	CYP2C9	CYP2D6	CYP3A4
Clopidogrel + Ilaprazole	-0.161	-0.04	0.955	-0.095	-0.475	-0.243	-0.714
Clopidogrel + Pantoprazole	-0.122	-0.089	0.929	-0.256	-0.594	-0.218	-0.772
Clopidogrel + Omeprazole	-0.137	-0.052	0.96	-0.177	-0.364	-0.168	-0.805
Clopidogrel + Esomeprazole	-0.137	-0.052	0.96	-0.177	-0.364	-0.168	-0.805
Clopidogrel + Lansoprazole	-0.212	0.063	0.962	-0.111	-0.556	-0.353	-0.859
Clopidogrel + Rabeprazole	-0.148	0.055	0.959	-0.045	-0.552	-0.314	-0.848
Clopidogrel + Dexlansoprazole	-0.212	0.063	0.962	-0.111	-0.556	-0.353	-0.859
Clopidogrel + Dexrabeprazole	-0.148	0.055	0.959	-0.045	-0.552	-0.314	-0.848

The results indicates that all the proton pump inhibitors that has been evaluated in the study found to have interaction with the clopidogrel. The pharmacokinetic interaction between the clopidogrel and PPIs are mediated by CYP2C19. The lowest value for pantoprazole and ilaprazole shows they are comparatively having lesser effect of interaction.

## Discussion

The influence of various PPI on clopidogrel efficacy has been demonstrated in *in vitro* and *in vivo* studies. Ilaprazole, a potent and safe PPI, has not been tested for drug interaction with clopidogrel until now. This study aims to predict the DDI between the above combination using computational models. The developed models, which enable the prediction of several DDIs parameters such as risk level, severity and overlapping of P450 activities, are freely accessible through the Way2Drug.com, DDIs web service.

DDI prediction mediated by CYP450 isoforms has been evaluated for Ilaprazole and clopidogrel combination and estimated ΔP (probability of interaction) value for major seven cytochrome P450 isoforms. Positive Δp-values for CYP2C19 (0.955) indicate that it is involved in the drug interaction of Ilaprazole and clopidogrel. In contrast, negative Δp-values for other DMEs, CYP1A2, CYP2B6, CYP2D6, CYP2C8, CYP2C9, CYP2C19 and CYP3A4, indicate that they are not involved in drug interaction of ilaprazole and clopidogrel biotransformation (Figure 1).

The CYP2C19 genetic polymorphism does not significantly influence ilaprazole due to its pharmacokinetic variability compared with conventional PPIs [17].

Implementing PASS algorithm and PoSMNA descriptors, specifying the roles of compounds as substrate, inhibitor or inducer. The probability of its role is given as Δp-value estimated for clopidogrel and Ilaprazole (Table 1). The results indicated that clopidogrel has substrate role in CYP2C19, CYP2C9 and CYP3A4; it has a effect of inhibition in CYP2C9 and CYP2C19. Ilaprazole was found to be a substrate of all the seven major CYP450 isoforms, and it has an inhibitory effect on CYP2C19, CYP3A4, CYP2C9 and CYP2D6.

DDI prediction for clopidogrel and various proton pump inhibitors mediated by CYP450 isoforms has been estimated (Table 2). The findings indicate that drug–drug interaction for proton pump inhibitors and clopidogrel occurs at the level of metabolism mediated by CYP2C19. Other cytochrome P450 isoforms with negative ΔP-values show that those enzymes are not involved in drug interaction [18].

The results of *in silico* analysis findings indicate that there is a possibility of CYP2C19 mediated pharmacokinetic interaction between clopidogrel and Ilaprazole, but the probability of occurrence of DDI with ilaprazole is lesser for clopidogrel when compared with other PPIs except pantoprazole. Prescribing clopidogrel with other CYP2C19 inhibitors along with PPI may further potentiated the DDI between the drugs.

The maximum Interaction CYP2C19 ΔP-value was found in the order of lansoprazole and dexlansoprazole >omeprazole >esomeprazole >rabeprazole >dexrabeprazole and rabeprazole >ilaprazole >pantoprazole. Therefore, the DDI mediated by CYP2C19 is minimal for pantoprazole and Ilaprazole coadministered with clopidogrel.

Since CYP2C19 metabolizes omeprazole and its stereo-isomer esomeprazole exclusively, it has the highest potential for drug interactions. Although CYP2C19 also metabolizes rabeprazole and lansoprazole/dexlansoprazole, they have a strong affinity for CYP3A4. With these agents, interactions seem less significant, maybe because of this distinction. While pantoprazole has the lowest potential for cytochrome induction or inhibition among the benzimidazoles, as it is largely degraded via CYP2C19 O-demethylation and sulfate conjugation.

A major factor in the pathophysiology of ACS patients who are admitted to the hospital due to a recurrence of cardiovascular events is elevated platelet aggregation and the thrombus formation. Consequently, lowering the high morbidity and death rate associated with this requires the use of safe and efficient antiplatelet medication. Due to a possible decrease in efficacy in these patients, the FDA has issued a boxed warning for CYP2C19 poor metabolizers; however, multivariate analysis results indicate that other factors, such as age, sex, obesity, concurrent diseases and drug–drug interactions, may also play a role in the overall between-subject variability in treatment response. Juurlink *et al.* revealed a higher risk of reinfarction and a decrease in the therapeutic effects of clopidogrel in patients undergoing clopidogrel therapy with a PPI other than pantoprazole [19–21].

Consequently, studies suggests that coadministering an inhibitor or inductor of CYP3A4 or other P450 enzymes will not appreciably impact the exposure of ilaprazole in humans. Studies also revealed that polymorphism of these metabolizing enzymes will not be significantly affects the concomitant drugs. The personalize clopidogrel treatment by identification of the *CYP2C19 LoF* alleles and assessment of drug–drug interaction will be helpful to reduce the risk of reinfarction and mortality rate due to therapeutic failure of clopidogrel [22,23].

## Conclusion

Computational methods are helpful in the drug development process as well as in clinical aspects for predicting, detecting, and declaring DDIs. PPIs have short plasma half-lives but might competitively inhibit CYP2C19, thereby affecting the clinical efficacy of clopidogrel. Ilaprazole overcomes the limitations of other PPIs related to CYP2C19, and having longer half-life with better gastric acid suppression. The drug interactions on the level CYP450 have been predicted for ilaprazole and clopidogrel, and found that it has also been carried out by CYP2C19 metabolic enzyme. Compared with other PPIs, Pantoprazole and Ilaprazole were found to have a low probability of CYP2C19 inhibition as per *in silico* results. There is insufficient evidence regarding which PPI among pantoprazole and ilaprazole is least likely to interact when coadministered with clopidogrel. Since Ilaprazole has pharmacokinetic variability, further *in vivo* and *in vitro* studies are required on the ilaprazole and clopidogrel combination to assess the effect of drug–drug interaction.
